# The combination of endobronchial elastography and sonographic findings during endobronchial ultrasound‐guided transbronchial needle aspiration for predicting nodal metastasis

**DOI:** 10.1111/1759-7714.13186

**Published:** 2019-09-01

**Authors:** Taiki Fujiwara, Takahiro Nakajima, Terunaga Inage, Yuki Sata, Yuichi Sakairi, Hajime Tamura, Hironobu Wada, Hidemi Suzuki, Masako Chiyo, Ichiro Yoshino

**Affiliations:** ^1^ Department of General Thoracic Surgery Chiba University Graduate School of Medicine Chiba Japan

**Keywords:** Elastography, endobronchial ultrasound‐guided transbronchial needle aspiration (EBUS‐TBNA), lung cancer, mediastinal and hilar lymph nodes, sonographic features

## Abstract

**Background:**

During endobronchial ultrasound‐guided transbronchial needle aspiration (EBUS‐TBNA), the sonographic findings of B‐mode imaging, as well as endobronchial elastography, can be obtained noninvasively and used for the prediction of nodal metastasis.

**Methods:**

Patients with lung cancer or suspected lung cancer who underwent EBUS‐TBNA were recorded prospectively and reviewed retrospectively. Both the B‐mode sonographic and elastographic findings were independently evaluated for each lymph node. The sonographic features were classified according to previously published criteria. If oval shape, indistinct margins, homogenous echogenicity, and the absence of coagulation necrosis sign were all observed by B‐mode imaging, then the lymph node was judged to be benign by sonographic imaging. In addition, if the stiffer area comprised more than 31% of the entire lymph node area, then the lymph node was judged to be malignant by elastography. We compared the results of these imaging‐based predictions with the pathological diagnoses.

**Results:**

The prevalence of nodal metastasis was 78/228 (34.2%). B‐mode sonography predicted 95.8% of benign lymph nodes, and elastography predicted 72.1% of malignant lymph nodes. By combining the two modalities, 59 of 71 (83.1%) lymph nodes judged as malignant by both analyses were pathologically proven to be malignant, and 101 of 105 (96.2%) lymph nodes judged as benign by both analyses were pathologically proven to be benign.

**Conclusion:**

The combination of elastography and sonographic findings showed good sensitivity and a high negative predictive value, which may facilitate selecting the most suspicious lymph nodes for biopsy.

**Key points:**

- **Significant findings of the study.**
  The combination of endobronchial elastography and sonography resulted in a higher diagnostic yield than either modality alone for predicting benign and malignant lymph nodes in patients with lung cancer.
- **What this study adds.**
  The combination of endobronchial elastography and sonography will help clinicians identify the most suspicious lymph nodes for puncturing during EBUS‐TBNA, which may improve the efficiency of EBUS‐TBNA.

## Introduction

Endobronchial ultrasound‐guided transbronchial needle aspiration (EBUS‐TBNA) is a minimally invasive modality for nodal staging in patients with lung cancer.^1^, ^2^, ^3^ Following the current clinical guideline, EBUS‐TBNA is recommended over surgical staging as the first step for mediastinal staging.^4^ During the staging procedure, it is common to encounter multiple lymph nodes within the same station. In such cases, the sonographic features of EBUS can be evaluated to determine the most suspicious lymph nodes at highest priority for biopsy. The utility of six distinctive sonographic features for predicting nodal metastasis was reported previously during EBUS‐TBNA.^5^ Recent advances in ultrasound scanners have enabled visualization of the relative stiffness within the region of interest by elastography, and endobronchial elastography can also be used to predict nodal metastasis during EBUS‐TBNA.^6^, ^7^, ^8^ Whereas the diagnostic value of each imaging modality has been evaluated independently, the efficacy of the combination of different imaging modalities is unclear.

In this study, we assessed the utility of elastography and B‐mode sonography during EBUS for the prediction of benign and malignant lymph nodes.

## Methods

### Study design

The data from sonographic and elastographic images during EBUS‐TBNA as well as the pathological diagnoses were prospectively recorded for the purpose of this study (approved by the Institutional Review Board by Chiba University Graduate School of Medicine, No. 1973). These prospective data collection were registered at the UMIN Clinical Trials Registry (UMIN ID: 000016106). The correlation between imaging findings and the pathological diagnosis was evaluated retrospectively in 122 patients with lung cancer or suspected of having lung cancer at Chiba University Hospital between November 2014 and April 2016. This retrospective study was approved by the Institutional Review Roard of Chiba University Graduate School of Medicine, No. 3200.

### Indications for EBUS‐TBNA

The lymph node location and cancer stage were defined based on the seventh edition of the TNM classification for lung cancer.^9^ All patients underwent contrast‐enhanced chest computed tomography (CT) first, and lymph nodes >10 mm in the short axis were judged to be enlarged. In addition, ^18^F‐fluorodeoxyglucose‐positron emission tomography (FDG‐PET)‐CT was also performed, and lymph nodes with a maximum standard uptake value (SUV_max_) >2.5 were deemed suspicious for metastasis. The indications for EBUS‐TBNA followed the ACCP guidelines.^4^ Elevated serum tumor marker levels (carcinoembryonic antigen >5.2 ng/mL) were also an indication for EBUS‐TBNA at our institute.

### EBUS‐TBNA procedure

EBUS‐TBNA was performed under local anesthesia with moderate conscious sedation using midazolam. A convex probe EBUS (BF‐UC260FW) and ultrasound scanner at a frequency of 10 MHz (EU‐ME2 Premier Plus), together with a dedicated 22‐G needle (NA‐201SX‐4022), were used in this study (Olympus, Tokyo, Japan). Before elastography, conventional sonographic features (B‐mode) were imaged. Both sonographic and elastographic images in JPEG format were obtained at the maximum diameter of the lymph nodes and for elastography at the vertex of the strain curve. In addition, all EBUS procedures, including ultrasound observations and TBNA, were recorded as video clips in MPEG‐4 format. EBUS‐TBNA was performed by board‐certified bronchoscopists (Taiki Fujiwara, Takahiro Nakajima, Inage Terunaga, Yuki Sata, and Yuichi Sakairi) or under their supervision according to a previously described method.^1^, ^5^ Rapid onsite cytological evaluation was used to confirm adequate sampling.^10^

### Review of EBUS image findings

The ultrasound images including B‐mode and elastography were reviewed by two individuals (T.F. and T.N.) independently who were blinded to the pathological diagnosis. The video clip was also reviewed to confirm the features of the B‐mode sonographic and elastographic images. The image of the lymph node at the largest diameter was used as the representative image.

The sonographic features of the lymph nodes were evaluated first, based on previously published EBUS B‐mode image classification criteria^5^ as follows: (i) size, (ii) shape, (iii) margin, (iv) echogenicity, (v) presence of a central hilar structure (CHS), and (vi) presence of coagulation necrosis sign (CNS). The representative B‐mode imaging features are shown in Figure S1. For classification of the sonographic features, the lymph nodes were judged as benign when the following four findings were all negative: round shape, distinct margins, heterogeneous echogenicity, and presence of a CNS. The lymph nodes were judged as malignant when any of those four findings was positive.

Elastographic images were recorded during EBUS‐TBNA and evaluated separately after EBUS‐TBNA using the image analysis software program Image J 1.45s (National Institutes of Health, Bethesda, MD, USA). Blue areas on elastography, which were considered stiffer than others, reflected cancer metastasis within the lymph node. As reported previously, the stiff area ratio was calculated and if the ratio exceeded 31%, the lymph node was considered malignant.^7^

### Correlation between sonographic features and pathological diagnosis

The sonographic features on B‐mode imaging and the stiff area ratio as determined by elastography were collated with the pathological results obtained by EBUS‐TBNA. In patients with malignant lymph nodes, the final diagnosis was made based on the pathological results of EBUS‐TBNA or surgical confirmation. In patients with benign lymph nodes, the diagnosis was based on surgical confirmation by lymph node dissection, or the results of a clinical follow‐up (for at least 12 months) evaluation demonstrating a lack of clinical or radiological disease progression.

### Statistical analysis

The sensitivity, specificity, positive predictive value, negative predictive value, and diagnostic accuracy were calculated by the standard definitions. We used the Stat View for Windows software program, version 5 (SAS Institute, Cary, NC, USA) for statistical analysis.

## Results

### Patients

The characteristics of the patients are summarized in Table 1. The patients comprised 94 men and 28 women, whose average age was 68.4 years. The reasons for undergoing EBUS were lung cancer diagnosis or staging in 73, evaluation of lung cancer recurrence in 43, and diagnosis of extrathoracic lymph node malignancy in six. A total of 228 lymph nodes were analyzed, and the numbers of lymph nodes in each station are summarized in Table 2. Radiologically‐suspected malignant nodes were frequently observed in station 4R and station 7 lymph nodes (143 [63%]) and were subjected to analysis. The final diagnosis included 78 malignant and 150 benign lymph nodes (Table 2). No patients had any severe complications related to EBUS‐TBNA.

**Table 1:** Characteristics of the EBUS‐TBNA enrolled patients

Case	*n* (N = 122)
Sex (male/female)	94/28
Age, average (range), years	68.4 (37―84)
Reason for EBUS	
Diagnosis or staging of lung cancer	73
Evaluation of lung cancer recurrence	43
Diagnosis of extrathoracic malignancy	6

EBUS, endobronchial ultrasound.

**Table 2:** Characteristics of the evaluated lymph nodes assessed by EBUS‐TBNA

Lymph node station	Numbers of lymph nodes (*N* = 228)
1R	1
2R/2L	16/2
3p	1
4R/4L	62/44
5	1
7	61
10	3
11s/11i/11(Lt.)	14/6/13
12	1
Tumor	2
Final diagnosis	
Malignant	78
Benign	150
Lymph node size on EBUS images	Average (range), mm
Malignant lymph nodes	15.6 (4.5―40)
Benign lymph nodes	7.8 (3.1―20.3)

Lt., Left.

### Sonographic features of B‐mode imaging during EBUS

**Figure 1:**
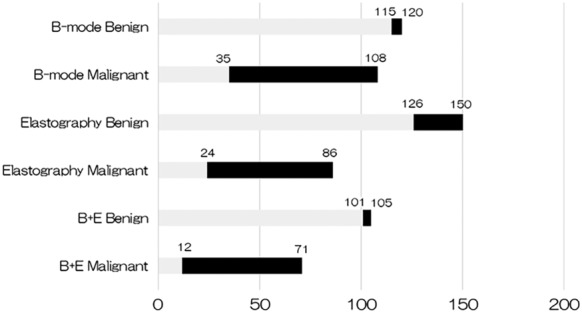
Comparison of the results between EBUS images and the final pathology. On B‐mode imaging, lymph nodes with an oval shape, indistinct margins, homogeneous echogenicity, and no coagulation necrosis sign tended to be benign. On elastography, lymph nodes with blue areas were considered to indicate relatively stiff areas with a tendency to be malignant. B+E = combination of elastography and sonographic findings. Benigin (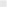); Malignant (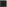).

The lymph nodes that were judged as benign based on their B‐mode sonographic features satisfied all four parameters (oval shape, indistinct margin, homogeneous echogenicity, and no CNS) according to a previous publication.^5^ In the present study, 115 of 120 (95.8%) lymph nodes judged as benign based on their sonographic features were ultimately proven to be benign, and 73 of 108 (67.6%) lymph nodes judged as malignant were ultimately proven to be malignant by pathology (Table 3, Fig 1).

**Table 3:** Diagnostic yields of EBUS for metastatic lymph nodes

	Sensitivity	Specificity	Positive predictive value	Negative predictive value	Diagnostic accuracy
Sonographic features	93.6	76.7	67.6	95.8	82.5
Elastography	72.1	84.0	72.1	84.0	79.7
Combination of elastography and sonographic findings	93.7	89.4	83.1	96.2	90.9

### Elastography during EBUS

The results of elastography for predicting nodal metastasis are shown in Figure 1, and the diagnostic yields are summarized in Table 3. Following imaging analysis, endobronchial elastography was performed, and 86 lymph nodes were judged to be malignant and 150 lymph nodes benign. The sensitivity and specificity of endobronchial elastography for predicting nodal metastasis were 72.1% (62/86) and 84.0% (126/150), respectively.

### Combination of elastography and sonographic findings for predicting malignancy

**Figure 2:**
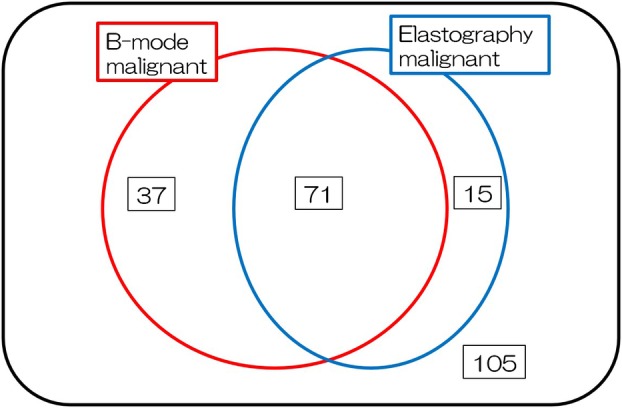
Venn diagram of the combination of sonographic and elastographic features. Both sonographic features and elastography indicated malignancy in 71 lymph nodes and benignancy in 105 lymph nodes.

Both the sonographic and elastographic features indicated malignancy in 71 lymph nodes with 105 benign lymph nodes (Fig 2). The sensitivity and specificity of the combination of endobronchial elastographic and sonographic findings categorization for the prediction of nodal metastasis were 83.0% (59/71) and 96.2% (101/105), respectively. The combination of elastography and sonography showed a higher diagnostic yield than either modality alone (Table 3). We defined a targeted lymph node size >10 mm in the short axis as suspicious malignancy in sonographic features. The sensitivity, specificity, and diagnostic accuracy of a lymph node size >10 mm alone for a diagnosing malignancy were 67.9%, 78.7%, and 75.0%, respectively. By adding elastographic findings to the lymph node size criterion, the sensitivity, specificity, and diagnostic accuracy improved to 89.4%, 95.1%, and 93.3% (Table 4).

**Table 4:** Diagnostic yields according to lymph node size criterion on EBUS improvement in the yield by addition of imaging

	Sensitivity	Specificity	Positive predictive value	Negative predictive value	Diagnostic accuracy
Lymph node size > 10 mm on EBUS	67.9	78.7	62.4	82.5	75.0
Combination of elastography and node size > 10 mm	89.4	95.1	89.4	95.1	93.3

## Discussion

During nodal staging by EBUS‐TBNA, we often encounter cases with multiple lymph nodes within the same nodal station. EBUS may aid in determining the most suspicious lymph nodes for sampling, resulting in a more efficient procedure. In this study, we combined elastography with sonography to predict nodal metastasis. Elastography had a high predictive ability for malignancy, and sonography had a high predictive ability for benignancy. Combining both analyses therefore resulted in an improved imaging accuracy with a higher diagnostic yield than either modality alone.

Endobronchial elastography using EBUS is a relatively new technology for endosonography capable of generating images that reveal the relative stiffness within the targeted area. EBUS can be used to predict benign or malignant lymph nodes.^6^, ^7^, ^8^ Izumo *et al*. classified the elastographic findings of EBUS into three categories: (i) predominantly nonblue, (ii) partially blue, and (iii) predominantly blue lymph nodes. Predominant blue staining was considered indicative of malignancy,^6^ and 94.6% of these lymph nodes were indeed confirmed to be malignant in their study. We calculated the ratio of the stiff area percentage within the lymph node to the entire lymph node area using ImageJ 1.45s software.^7^ The stiff area (blue area)/nodal area ratio was significantly greater for metastatic than benign lymph nodes (0.487 vs. 0.216). When the cutoff for the stiff area ratio was set to 0.311, the sensitivity and specificity for predicting malignancy were 0.81 and 0.85, respectively. These findings indicate that elastography is more accurate for predicting malignant lymph nodes than sonography.

**Figure 3:**
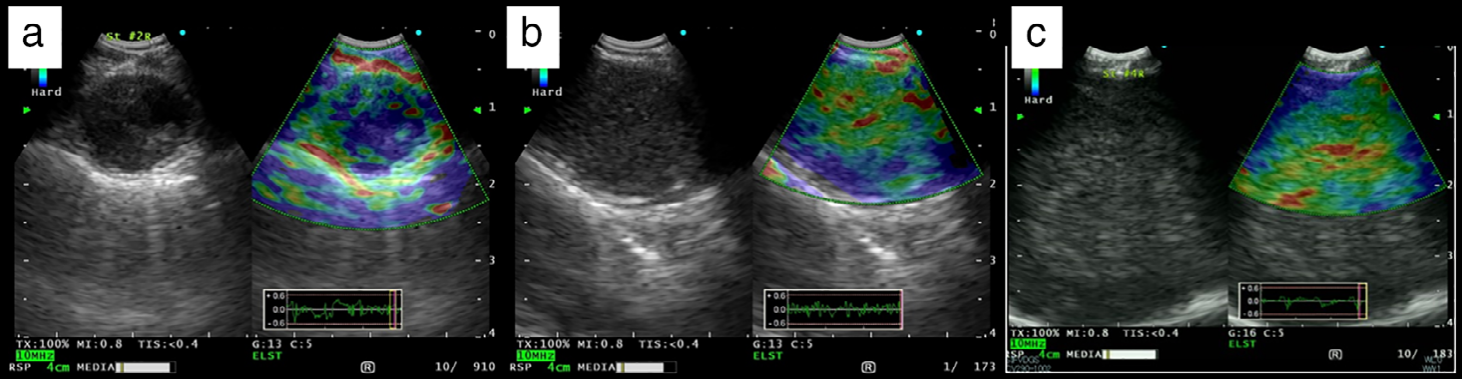
**(a**) Malignant lymph node pattern on elastography. The 2R lymph node was 13.6 mm in size, and abnormal FDG uptake on PET‐CT in the node indicated malignancy on elastography. The final diagnosis was malignancy. (**b**) Benign pattern lymph node pattern on elastography. The 3p lymph node was 17.6 mm in size, and abnormal FDG uptake on PET‐CT in the node indicated benignancy on elastography. The final diagnosis was benign. (**c**) False‐negative case. The 4R lymph node was 34.5 mm in size, and abnormal FDG uptake on PET‐CT in the node indicated benignancy on elastography. However, the final diagnosis was malignancy.

However, 24 false‐negative lymph nodes were detected by elastography alone in this study. Seven of these 24 lymph nodes had a necrotic component within the lymph node and apparent relative softness on elastography. In such cases, it was difficult to obtain an adequate diagnosis by elastography alone, but also taking into account the sonographic features allowed us to determine the diagnosis. Some representative cases are shown in Figure 3. In Figure 3c, necrosis was noted along with malignant cells during EBUS‐TBNA. The necrotic component tended to show relative softness on elastography. In such cases, considering the sonographic features can aid the diagnosis. Seven false‐negative cases that appeared benign based on elastography but malignant‐based on sonography had necrosis.

We previously reported the utility of EBUS findings for staging lung cancer.^5^ Regarding the sonographic features, when four independent predictive factors (oval shape, indistinct margin, homogeneous echogenicity, and absence of CNS) were considered indicators of benignancy, 96.0% of lymph nodes were ultimately deemed benign, whereas lymph nodes showing any one of those four findings (indicating malignancy) were ultimately deemed malignant in 42.9% of cases.^5^ While EBUS sonographic findings can help identify benign lymph nodes, firm classification of B‐sonographic findings requires substantial training and experience.^11^ Furthermore, the use of sonographic findings alone for predicting malignancy in lymph nodes represents a challenge to clinicians.

The major limitations of this study were that it was conducted at a single institution, and the results were based on a small number of samples. Multicenter prospective trials will be needed to confirm the results of this study in the future.

In conclusion, the combination of endobronchial elastography and sonographic findings resulted in a higher diagnostic yield than either modality alone. This knowledge will help clinicians identify the most suspicious lymph nodes for puncturing during EBUS‐TBNA, which may improve the efficiency of EBUS‐TBNA procedures.

## Disclosure

Takahiro Nakajima and Taiki Fujiwara received honoraria and lecture fees from Olympus Medical Systems for EBUS‐TBNA training courses. These sponsors had no role in the study design, study execution, data collection, data management or interpretation, preparation, review or approval of the report.

## Supporting information


**Figure S1** Representative B‐mode imaging features.
