# Supplementary figures and images for: The combination of endobronchial elastography and sonographic findings during endobronchial ultrasound‐guided transbronchial needle aspiration for predicting nodal metastasis
Source: Thorac Cancer. 2019 Sep 1;10(10):2000–5. doi: 10.1111/1759-7714.13186 (PMC6775026; doi:10.1111/1759-7714.13186)

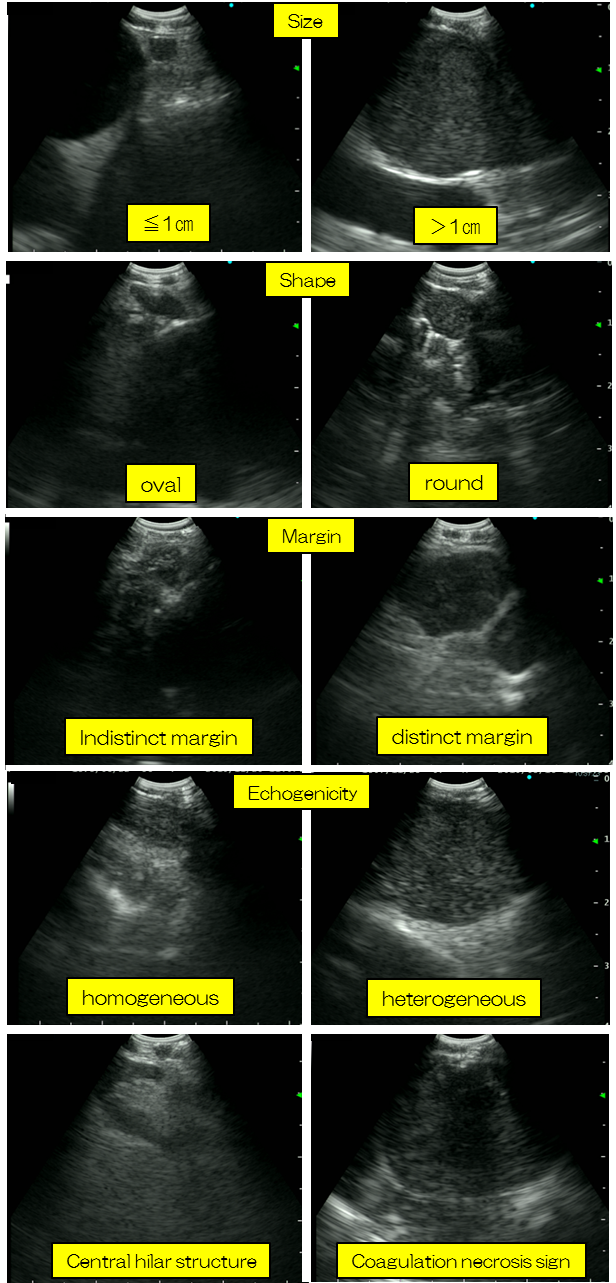

Supplement: Supplementary file 1 — Figure S1 Representative B‐mode imaging features. [file TCA-10-2000-s001.tif]
